# From Genome to Structure and Back Again: A Family Portrait of the Transcarbamylases

**DOI:** 10.3390/ijms160818836

**Published:** 2015-08-12

**Authors:** Dashuang Shi, Norma M. Allewell, Mendel Tuchman

**Affiliations:** 1 Center for Genetic Medicine Research, Children’s National Medical Center, the George Washington University, Washington, DC 20010, USA; E-Mail: mtuchman@childrensnational.org; 2 Department of Integrative Systems Biology, Children’s National Medical Center, the George Washington University, Washington, DC 20010, USA; 3 Department of Cell Biology and Molecular Genetics, College of Computer, Mathematical, and Natural Sciences, University of Maryland, College Park, MD 20742, USA; E-Mail: allewell@umd.edu; 4 Department of Chemistry and Biochemistry, College of Computer, Mathematical, and Natural Sciences, University of Maryland, College Park, MD 20742, USA

**Keywords:** transcarbamylase, pyrimidine biosynthesis, arginine biosynthesis, arginine deiminase pathway, agamatine deiminase pathway, viomycin biosynthesis, zwittermicin A biosynthesis, padanamide biosynthesis

## Abstract

Enzymes in the transcarbamylase family catalyze the transfer of a carbamyl group from carbamyl phosphate (CP) to an amino group of a second substrate. The two best-characterized members, aspartate transcarbamylase (ATCase) and ornithine transcarbamylase (OTCase), are present in most organisms from bacteria to humans. Recently, structures of four new transcarbamylase members, *N*-acetyl-l-ornithine transcarbamylase (AOTCase), *N*-succinyl-l-ornithine transcarbamylase (SOTCase), *ygeW* encoded transcarbamylase (YTCase) and putrescine transcarbamylase (PTCase) have also been determined. Crystal structures of these enzymes have shown that they have a common overall fold with a trimer as their basic biological unit. The monomer structures share a common CP binding site in their N-terminal domain, but have different second substrate binding sites in their C-terminal domain. The discovery of three new transcarbamylases, l-2,3-diaminopropionate transcarbamylase (DPTCase), l-2,4-diaminobutyrate transcarbamylase (DBTCase) and ureidoglycine transcarbamylase (UGTCase), demonstrates that our knowledge and understanding of the spectrum of the transcarbamylase family is still incomplete. In this review, we summarize studies on the structures and function of transcarbamylases demonstrating how structural information helps to define biological function and how small structural differences govern enzyme specificity. Such information is important for correctly annotating transcarbamylase sequences in the genome databases and for identifying new members of the transcarbamylase family.

## 1. Introduction

**Figure 1:**
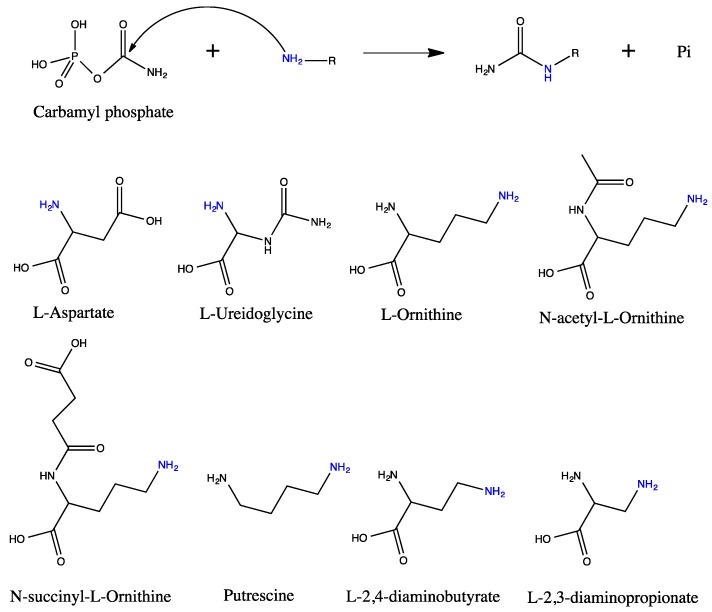
Schematic drawing of the carbamyltransferase reactions and the structures of its substrates. Carbamyltransferase catalyzes the transfer of a carbamyl group from carbamyl phosphate to the amino group (colored as blue) of the second substrate. The forward reaction is catalyzed by anabolic enzymes and the reverse reaction by catabolic enzymes. The forward reaction is kinetically favored.

The transfer of a carbamyl group from carbamyl phosphate (CP) to a nitrogen atom of another molecule is catalyzed by a family of enzymes termed transcarbamylases (Figure 1) of which aspartate transcarbamylase (ATCase) and ornithine transcarbamylase (OTCase) are the best-known members. ATCase catalyzes the first reaction in the *de novo* pyrimidine biosynthetic pathway, transferring of a carbamyl group from CP to l-aspartate to form *N*-carbamyl-l-aspartate [1]. ATCase is a ubiquitous enzyme which is present in almost all organisms, but with various quaternary structures in different organisms. Prokaryotic ATCases have three major types of quaternary structure. One type is a dodecameric holoenzyme, consisting of a complex of a single ATCase catalytic subunit with a single active or inactive dihydroorotase (DHOase) [2,3,4]. The second type is also dodecameric, but consists of two catalytic trimers linked by three regulatory dimers which may be either separated [5] or fused together [6]. A third type has only a catalytic trimer and is insensitive to allosteric effectors [7]. Two types of eukaryotic ATCases are known. Plants have a catalytic trimer similar to the third type of prokaryotic ATCase, but are sensitive to allosteric effectors [8]. In animals and the slime mould *Dictyostellium discoideum*, ATCase fuses with carbamyl phosphate synthetase 2 (CPS2) and an active DHOase to form a multifunctional polypeptide termed CAD (CPS2-ATCase-DHOase) [9]. CAD-like proteins occur also in fungi, but the DHOase domain is catalytically inactive [10]. Despite the variations in quaternary structure, the functional unit of all ATCases consists of a catalytically active homotrimer.

OTCase is also a ubiquitous enzyme that exists in nearly all organisms. Two types of OTCases are known: anabolic and catabolic. While anabolic OTCases catalyze the carbamylation of l-ornithine to form citrulline within the arginine biosynthetic pathway in lower organisms and the urea cycle in mammals [11,12], catabolic OTCases promote the reverse reaction within the arginine deiminase pathway which degrades arginine to ornithine, and produces ornithine and CP from citrulline through phosphorolysis [13,14]. Catabolic OTCases are found only in lower microorganisms, which use arginine as an energy source to generate ATP. The functional unit of anabolic OTCases is generally a trimer with the following exceptions. The OTCases from two actinomycetes (*Streptomycetes clavuligerus* and *Nocardia lactamdurans*) are hexameric enzymes [15] that possess both anabolic and catabolic functions. The anabolic OTCase from the thermophilic bacterium, *Pyrococcus furiosus*, is a dodecameric enzyme, with increased thermal stability [16]. Similarly, most catabolic enzymes are dodecamers that are sensitive to allosteric effectors [12,17,18,19], with some exceptions; for example, the catabolic OTCase from *Lactobacillus hilgardii* was reported to be a hexamer [20].

Three additional transcarbamylases were identified recently in a few of bacteria. A novel *N*-acetyl-l-ornithine transcarbamylase (AOTCase) that catalyzes the carbamylation of *N*-acetyl-l-ornithine to form *N*-acetyl-l-citrulline in a modified arginine biosynthetic pathway was identified in *Xanthomonas campestris* and other eubacteria [21,22]. The structure determination of AOTCase led to the identification of another novel transcarbamylase, *N*-succinyl-l-ornithine transcarbamoylase (SOTCase), in *Bacteroides fragilis* [23]. Functional assignment was confirmed by catalytic studies and structure determination [24]. The presence of the latter enzyme suggests that *B. fragilis* and some other bacteria with this unique protein have a novel arginine biosynthetic pathway that uses succinylated derivatives as intermediates [24]. AOTCase and SOTCase are distinguished from one another by three amino acid substitutions [25].

The primary sequence of putrescine transcarbamylase (PTCase) is closely related to OTCase, enabling it to be identified in genomic data and by phylogenetic analysis [26]. It is involved in the catabolism of the polyamine agmatine in the agmatine deiminase pathway found in several Gram-positive bacteria [27]. Most PTCases have been erroneously annotated as OTCases because of their high sequence similarity [26]. The assignment of PTCase of *Enterococcus faecalis* was recently confirmed enzymatically and structurally [28,29]. The liganded and unliganded structures indicate that the active subunit is trimeric, similar to anabolic OTCase, AOTCase and SOTCase [29,30].

Among more than 40,000 transcarbamylase sequences found in the uniprot (www.uniprot.org) database, there are still a number of sequences that form independent clades that are distantly related to the above transcarbamylases in the phylogenetic tree; the functions and pathways of these transcarbamylases remain unknown [26]. Using the reaction module concept and bioinformatics analysis, a novel transcarbamylase, ureidoglycine transcarbamylase (UGTCase), was recently identified in the purine degradation pathway in *Rubrobacter xyaniphilus* [31]. The sequences of UGTCase are quite similar to ATCase and they have been annotated as a pseudo ATCase in the databases. The structure of one particular transcarbamylase of unknown function, *ygeW* encoded transcarbamylase (YTCase), was recently determined, revealing a canonical trimeric tertiary structure, but a very different active site structure [32].

New transcarbamylases that catalyze the carbamylation of l-2,3-diaminopropionate (Dap) to form β-ureidoalanine (Uda) emerged from the characterization of the biosynthetic gene cluster for zwittermicin A in *Bacillus cereus* and the viomycin biosynthetic gene cluster in *Streptomyces lividans* [33,34]. Homologous genes can be identified in most *Streptomyces* genera. Similarly, another new transcarbamylase that catalyzes the carbamylation of l-2,4-diaminobutyrate (Dab) to l-2-amino-4-ureidobutyrate (Aub) has been identified in *Streptomyces* sp. RJA2928 from the analysis of a biosynthetic gene cluster in padanamides. It will be interesting to investigate how subtle structural differences in these transcarbamylase members confer specificities for ligands that have side-chains that are one or two carbons shorter than l-ornithine.

Structures and mechanisms of ATCase have recently been reviewed [35,36]. The present review focuses on a comparison of all known and unknown members of the transcarbamylase family. Recent progress in crystallographic analyses has provided new insights into the relationship among the structures, functions and sequences that will aid in establishing correct annotations of transcarbamylase sequences in genomic databases.

## 2. Structures Deposited in the Protein Data Bank (PDB)

As of the end of 2014, 138 three-dimensional structures of transcarbamylase superfamily members have been deposited in the PDB. These structures, together with their source, ligands and PDB ID are summarized in Supplementary Table S1.

*Aspartate transcarbamylase*—Of the 81 structures deposited in the PDB, 64 are of *E. coli* ATCase complexed with different ligands and various mutant forms. Thus, *E. coli* ATCase is one of the best structurally characterized enzymes. Most of these structures are of the dodecameric holoenzyme, which consists of two catalytic trimers and three regulatory dimers, and is sensitive to allosteric effectors [5,37,38,39]. Three are structures of the isolated catalytic trimer [40,41]. Seventeen structures are from organisms other than *E. coli*, six are from the hyperthermophilic archaeons, *Pyrococcus abyssi* [42], *Sulfolobus acidocaldarius* [43,44] and *Methanococcus jannaschii* [45,46,47], and one is from the γ-division of proteobacteria *Moritella profunda*, a psychrophilic deep sea bacterium [48]. *S. acidocaldarius* ATCase was solved as the dodecameric holoenzyme, while the structure from *P. abyssi* is of the catalytic trimer complexed with the bisubstrate analogue, *N*-phosphonacetyl-l-aspartate (PALA). The structures of the catalytic trimer and regulatory dimer alone of *M*. *jannaschii* ATCase were also determined. The structure of *M.* *profunda* was determined in the T-state unliganded form.The only ATCase structure corresponding to a functional catalytic trimer *in vivo* is that of *Bacillus subtilis* [7,49]. Two structures of a prokaryotic ATCase from *Auifex aeolicus* that form a stable dodecameric holoenzyme with DHOase, were determined [50,51]. Only one eukaryotic ATCase structure, of *Trypanosoma cruzi*, has been determined (PDB code: 4IV5).

*Ornithine transcarbamylase*—Thirty-three OTCase structures from 18 different organisms have been determined. Most are from bacteria and archaea: three from *E. coli* [52,53,54], two from the γ-division of proteobacteria, *Pseudomonas aeruginosa* [55], two from *Mycobacterium tuberculosis* [56], two from the hyperthermophilic archaea, *Pyrococcus furiosus* [57], and three from the thermophilic cyanobacteria *Thermotoga maritima* and *Thermus thermophilus*. Fourteen structures represent anabolic OTCases while the two structures from *P. aeruginosa* represent catabolic OTCases. The biological subunit of the anabolic OTCases from *E. coli*, *M. tuberculosis*, humans and sheep is a trimer while those of the catabolic OTCase from *P. aeruginosa* and the anabolic OTCases from the hypertherphilic *P. furiosus* and *T. maritima* are dodecamers, in which four trimers form a tetrahedron with the concave faces of the trimers facing outwards. However, the OTCase from the thermophilic *T. thermophilus* appears to be a trimer. Among mammals, four OTCase structures from humans [21,58,59,60] and one from sheep [61], have been determined.

*N-acetyl-l-ornithine transcarbamylase*—Twelve structures of AOTCase from *X. campestris* were determined in complex with different ligands, including several structures of mutants [21,25,62], making AOTCase one of the best-characterized members of the transcarbamylase family.

*N-succinyl-l-ornithine transcarbamylase*—Four structures of *B. fragilis* SOTCase have been determined in different liganded states and with various mutations [23,24].

*Putrescine transcarbamylase—*Five structures of PTCase from *E. faecalis* with and without ligands have been determined that provide significant insight into its structure, function and mechanism [29,30].

*ygeW* encoded transcarbamylase of unknown function—Four structures of *E. coli* and *E. faecalis* YTCase have been determined. Although the structures clearly demonstrate that CP is the substrate for carbamylation, the second substrate and thus the biological function of this protein remain unknown [32].

## 3. Sequences of Transcarbamylases

In the NCBI genomic database, 13,608 bacterial, 533 archaeal, 30,677 fungal, two plant (*Arabidopsis thaliana*, *Oryza sativa*), two insect (*Apis mellifers* and *Drosophila melanogaster*), one fish (*Danio rerio*), one frog (clawed frog), one chicken (*Gallus gallus*) and nine mammalian (human, mouse, rat, cow, pig, dog, rabbit, guinea pig and chimpanzee) genomes are available for Blast searches of transcarbamylase sequences as of 2 January 2015. Two transcarbamylase sequences, one for ATCase and one for OTCase, are available for most species, including mammals. As indicated earlier, mammalian OTCase functions within the urea cycle, while ATCase is involved in the biosynthesis of pyrimidines. Mammalian ATCase sequences are usually fused to the sequences of CPS2 and DHOase to encode a polyfunctional protein termed CAD. Among invertebrates, most insects have ATCase, but do not have OTCase except for the honeybee, which has both anabolic and catabolic OTCases. Similarly, the nematode *Caenorhabditis briggsae* does not have an OTCase. However, the purple sea urchin does have an OTCase. Other urea cycle enzymes, including *N*-acetylglutamate synthase and arginase have also been identified in this organism, suggesting that it may have a functional urea cycle.

Most protozoa have only ATCase for synthesizing pyrimidines. However, five different transcarbamylase-like sequences from *Trichomonas vaginalis* G3 have been deposited in the database (XP_001315726, XP_001301097, XP_001326968, XP_1298740 and XP_1298741), none of which seems to be an ATCase. Two of them (XP_001315726 and XP_001301097) were annotated as OTCase, but have unusual DxxxSYH and NCLP motifs. Since no other genes in the arginine biosynthetic pathway, such as acetylglutamate kinase, argininosuccinate synthetase and lyase, were found, it is likely that these genes do not function as anabolic OTCases. Instead, enzymes for the arginine dihydrolase pathway, which converts arginine to ornithine with the generation of ATP, have been found, suggesting that these transcarbamylases likely function as catabolic OTCases [63]. The third sequence (XP_001326968) corresponds to YTCase, whose homologue sequence can be also identified in certain bacteria such as *E. coli*. The sequences XP_1298740 and XP_1298741 appear to be incomplete. If the stop codon TAA in XP_1298740 is changed to GAA for Glu, XP_1298740 and XP_1298741 will together encode a 417 amino acid full-length transcarbamylase that has 90.6% sequence identity to XP_00132968. It may be that the apparent stop codon is a sequencing error and that *Trichomonas vaginalis* G3 has two YTCases.

Most plants have two transcarbamylases, OTCase and ATCase. However, two OTCase isoenzymes were identified in the leaves of *Canavalia lineata*. Both can effectively use ornithine or canaline as a substrate, but one has higher *in vitro* ornithine-dependent activity while the other has higher canaline-dependent activity [64]. The sequences of these isoenzymes are very similar with 70% sequence identity. Canaline and canavanine, which are guanidooxy analogs of ornithine and arginine, respectively, are both nitrogen storage molecules in plants and are synthesized from homoserine using enzymes involved in the arginine biosynthetic pathway [65]. In the pea (*Pisum sativum* L.), two ATCase isomers with 83% sequence identity were identified [66].

Most fungal genomes contain two transcarbamylases, one for OTCase, and the other for ATCase, which usually fuses to CPS2 via an inactive pseudo-DHOase domain, although classified as a bifunctional protein [67,68].

The number of transcarbamylase-like sequences in bacteria and archaea varies significantly, ranging from zero to six. Some bacteria, such as *Helicobacter pylori*, contain only one transcarbamylase sequence corresponding to ATCase, but others have more than four transcarbamylase sequences. Six transcarbamylase sequences have been identified in *Nocardioides* sp. (strain BAA-499), which is able to assimilate vinyl chloride. Among them, a significant number of sequences do not have essential motifs of known transcarbamylases. These sequences may encode novel transcarbamylases the biological functions of which are still unknown.

**Figure 2:**
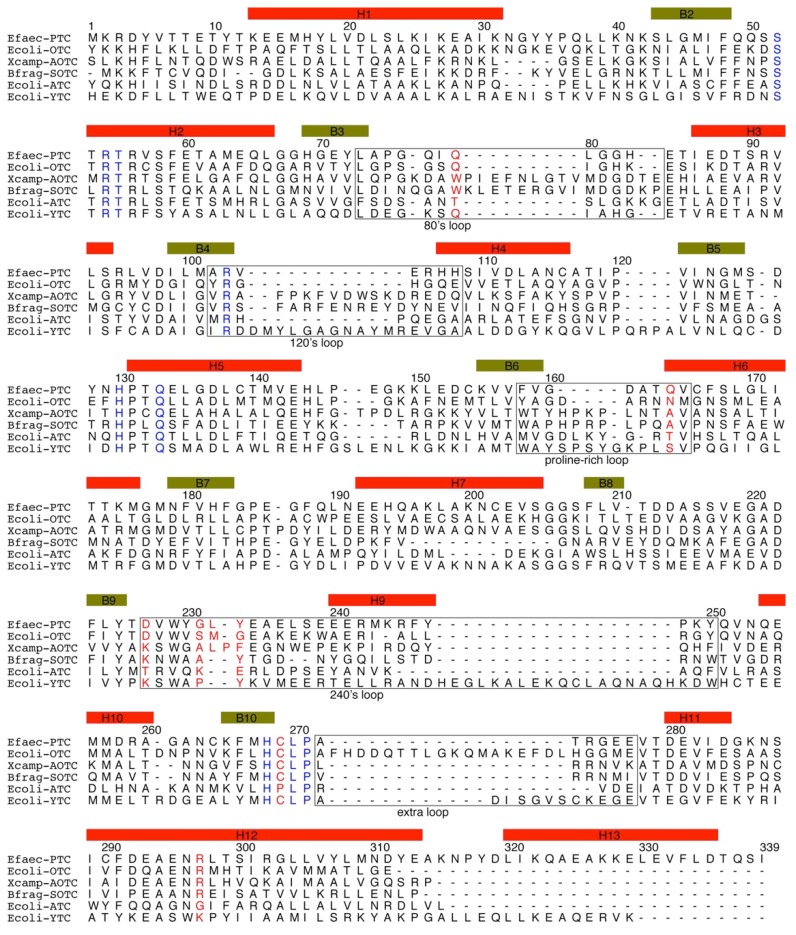
Sequence alignment of *Enterococus faecalis* PTCase, *Escherichia coli* OTCase, *Xanthomonas campestris* AOTCase, *Bacteroides fragilis* SOTCase, *E. coli* ATCase and *E. coli* YTCase. Sequence encoding secondary structure elements (based on the *E. faecalis* PTCase structure) are indicated by boxes in yellow-green (β-strand) and red (α-helix). The conserved motifs, SxRT, HPxQ and HxLP across transcarbamylase members are indicated in blue. Nonconserved residues, which might be involved in binding substrates, are indicated in red. The 80’s, 120’s, proline-rich, 240’s and extra loops are boxed.

The primary sequence alignment of selected transcarbamylase sequences from different members of the transcarbamylase family is shown in Figure 2. The greatest conservation across the superfamily is in three regions: the Fx(E/K/N/D/A/Q)xSxRT motif, the HPxQ motif and the HxLP motif. These three motifs define the common characteristics of the transcarbamylase family. Sequences in four loop regions, the 80’s loop, 120’s loop, proline-rich loop and 240’s loop, vary significantly among different transcarbamylase members.

## 4. Overview of the Structural Fold

**Figure 3:**
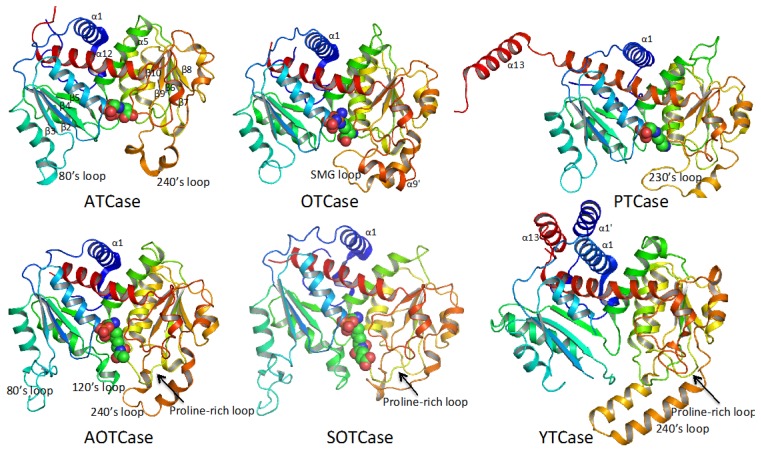
Ribbon diagram of the catalytic subunit of *Escherichia coli* ATCase, *E. coli* OTCase, *Enterococus faecalis* PTCase, *Xanthomonas campestris* AOTCase, *Bacteroidesfragilis* SOTCase and *E. coli* YTCase. The ribbons are colored in rainbow from blue (N-terminus) to red (C-terminus). The bound substrates or inhibitors are shown in space-filling models. The 3_1_ trefoil knots are formed by residues at the C-terminal end threading through the proline-rich loop (indicated by arrows) in AOTCase, SOTCase and YTCase.

Despite functional variations across the transcarbamylase superfamily, the protein topology of the catalytic subunit is quite similar as shown in Figure 3. The subunit structures of all transcarbamylase members can be divided into two domains: the CP domain and the second substrate-binding domain. Both domains have an αβα structure with a parallel β-sheet in the center and α helices on both sides. The two domains are linked by two α helices (helices 5 and 12 in *E. coli* ATCase). The fold of the CP domain in all known transcarbamylases is virtually identical consisting of five central β strands arranged in 1-5-4-2-3 topology. The five central β strands of the second substrate-binding domain of all transcarbamylases also have a common 8-7-6-9-10 topology. However, the second domains of ATCase, OTCase and PTCase are unknotted, while the second domains of AOTCase, SOTCase and YTCase contain a 3_1_ trefoil knot. The knot in these proteins requires many residues (85 residues in AOTCase, 70 residues in SOTCase and 124 residues in YTCase) at the C-terminal end to thread through the proline-rich loop [32] (Figure 3). The joint occurrence of the proline-rich loop and the knotted fold suggests that a proline-rich loop is a pre-requisite for knot formation.

**Figure 4:**
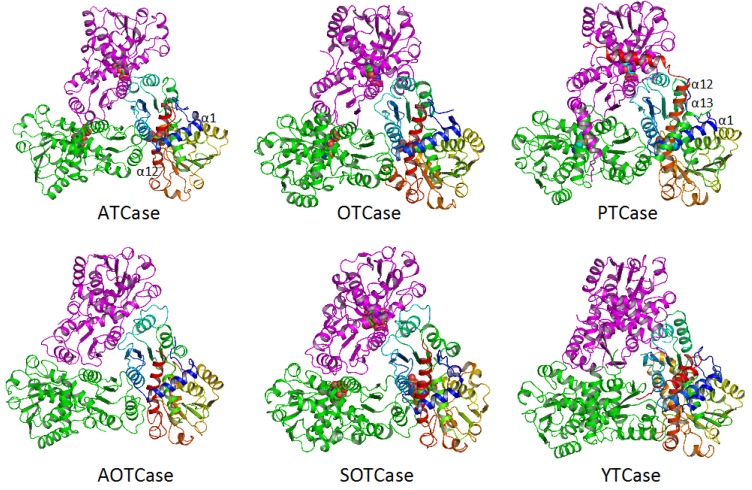
Ribbon diagram of the catalytic trimer of *Escherichia coli* ATCase, *E. coli* OTCase, *Enterococus faecalis* PTCase, *Xanthomonas campestris* AOTCase, *Bacteroides* *fragilis* SOTCase and *E. coli* YTCase, viewed down the three-fold axis. Different subunits are shown in different colors (rainbow, green and magenta, respectively). The bound substrates or inhibitors are shown as space-filling models.

Although all members of the transcarbamylase family have a similar fold, each member has its own distinctive features. The 240’s loop, a major recognition site for the second substrate, has different conformations in different members of the family. YTCase, in particular, has two extra helices in its equivalent 240’s loop. AOTCase and SOTCase have extended 80’s and 120’s loops relative to the unknotted transcarbamylase members, ATCase, OTCase and PTCase. The 80’s and 120’s loops in *E. coli* YTCase are disordered, probably because substrates are not present. Based on the sequence alignment (Figure 2), the conformation of the 80’s loop in YTCase should be very similar to that of ATCase and OTCase, while the 120’s loop would be expected to be similar to that of AOTCase and SOTCase. YTCase also has extra helices at both its N-terminal and C-terminal ends. These helices sit on helix 1, forming a three-helix bundle. PTCase’s special feature is an extra long helix at its C-terminal end, which extends to cover helix 1 of the adjacent subunit (Figure 4). This feature appears to be important in stabilizing the catalytic trimer and preventing formation of a larger oligomer [29,30].

**Figure 5:**
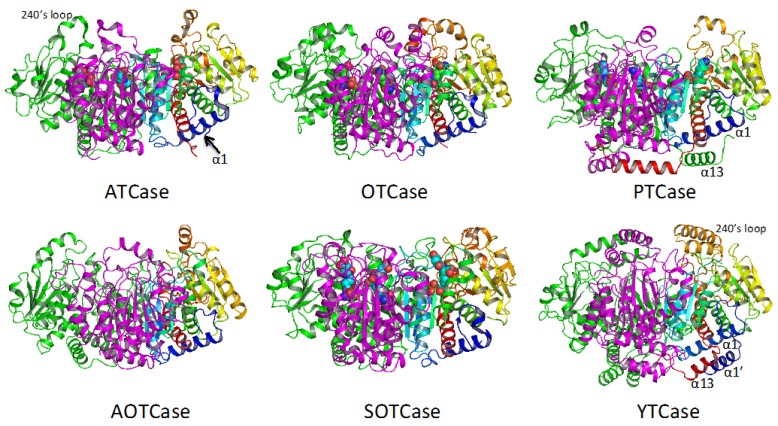
Ribbon diagram of the catalytic trimer of *Escherichia coli* ATCase, *E. coli* OTCase, *Enterococus faecalis* PTCase, *Xanthomonas campestris* AOTCase, *Bacteroides* *fragilis* SOTCase and *E. coli* YTCase, viewed perpendicular to the three-fold axis. Different subunits are shown in different colors (rainbow, green and magenta, respectively). The bound substrates or inhibitors are shown as space-filling models.

The basic catalytic unit for all transcarbamylase members is a trimer, even though most ATCases tend to form larger aggregates by fusing or binding to other enzymes with different catalytic activities. The trimer is shaped like a triangular cup with a radius of about 50 Å (Figure 4). The three N-terminal CP domains interact with each other close to the threefold axis, forming the bottom of the cup, while the three C-terminal domains protrude from the concave face to form the rim of the cup (Figure 5). In ATCase, OTCase, AOTCase and SOTCase, the N-terminal helix 1 (α1) which runs across helix 12 at about a 60° angle, forms the ridge of the convex face of the trimer. In PTCase, this helix is covered by the extra C-terminal helix (α13) from the adjacent subunit. In YTCase, two additional helices (α1′ and α13) sit on top of this helix. The 240’s loop, which is located at the concave face of the trimer, forms a cover that moves towards the active site during the catalytic reaction. In YTCase, because of the presence of two extra helices in the 240’s loop, the concave mouth is much smaller.

## 5. Active Site and Substrate Specificities

**Figure 6:**
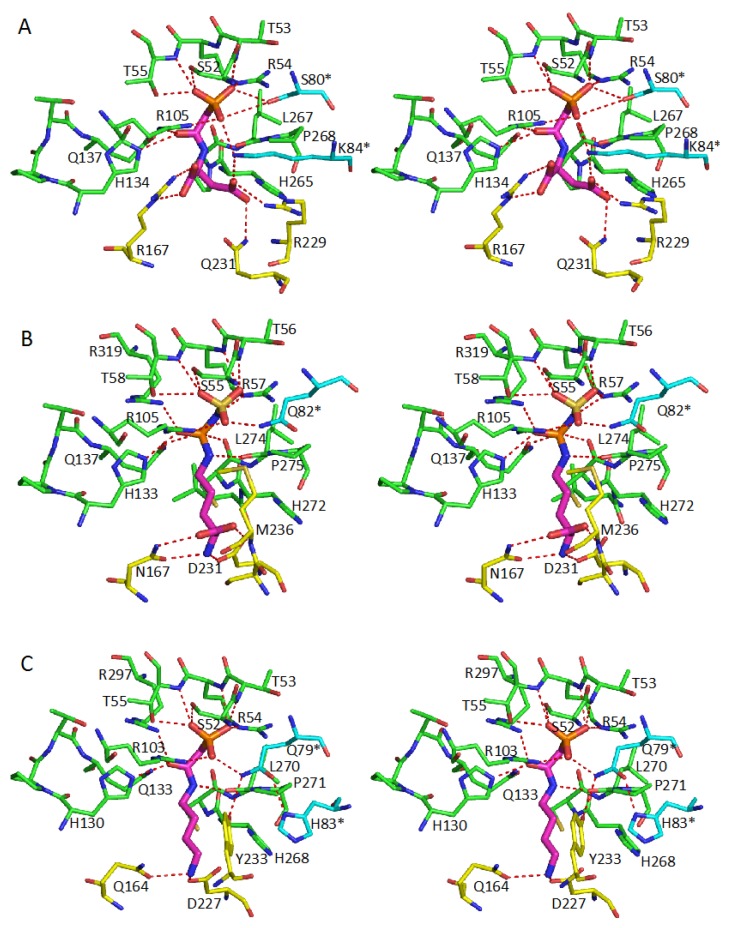
Stereo diagrams of the active sites of *Escherichia coli* ATCase (**A**); *E. coli* OTCase (**B**); and *Enterococus faecalis* PTCase (**C**). The residues involved in binding CP are shown as green sticks. The residues involved in binding the second substrate are indicated as yellow sticks. The residues from the adjacent subunit are indicated as cyan sticks. The bound substrates or inhibitor are shown as thick magenta sticks. The oxygen and nitrogen atoms are shown in red and blue sticks, respectively. The potential hydrogen bonding interactions are indicated by red dotted lines.

**Figure 7:**
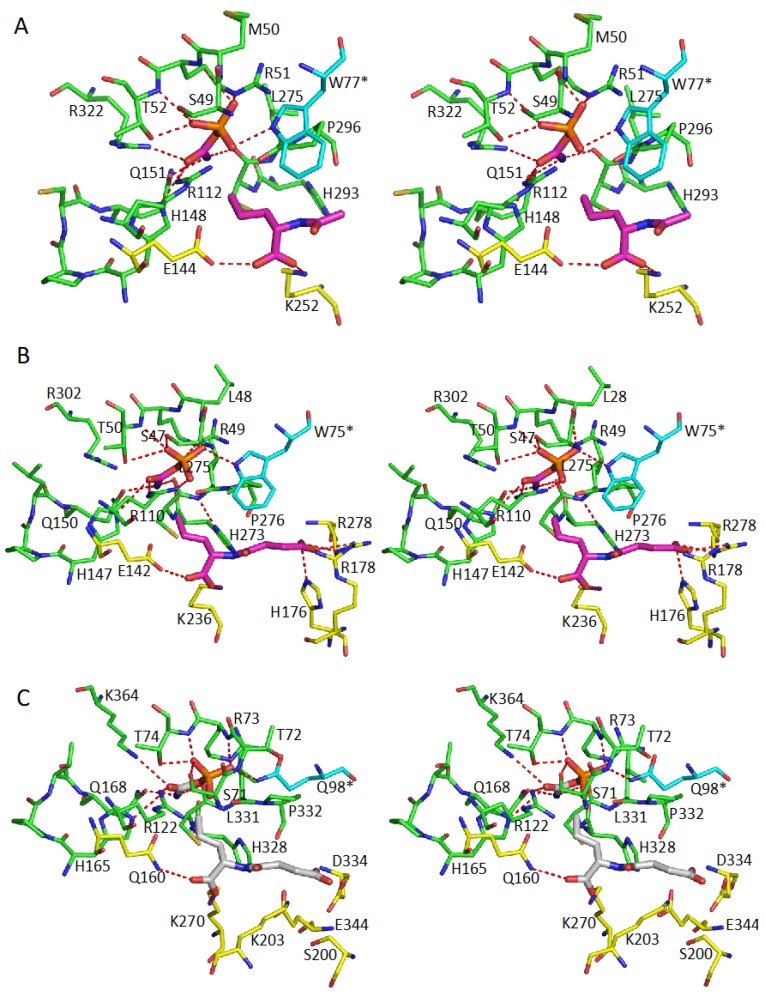
Stereo diagrams of the active sites of *Xanthomonas campestris* AOTCase (**A**); *Bacteroides* *fragilis* SOTCase (**B**); and *Escherichia coli* YTCase (**C**). The residues involved in binding CP are shown as green sticks. The residues involved in binding the second substrate are indicated by yellow sticks. The residues from the adjacent subunit are indicated by cyan sticks. The bound substrates or inhibitor are shown as thick magenta sticks. The residues in the original model (PDB code 3Q98) that are missing in YTCase were modeled based on OTCase *Vibrio vulnificus* (PDB code 4H31) and *Enterococus faecalis* YTCase (2YFK) using the IntFold server [69]. The CP and *N*-succinyl-l-norvaline in YTCase shown as grey sticks are not in the original model, but indicate the possible substrate binding site. The oxygen and nitrogen atoms are shown in red and blue sticks, respectively. The potential hydrogen bonding interactions are indicated by red dotted lines.

The active sites of all transcarbamylase members are located at the concave face of the trimer, in the cleft between the two domains and the interface between two subunits (Figure 4 and Figure 5). Since the active site involves residues from two adjacent subunits, it is not surprising that a trimer is the basic catalytic unit. In the CP binding site, the SxRT motif provides a major recognition site for the binding of the phosphate moiety of CP, while the HPxQ motif is the major site of interactions with the carbamyl moiety. Even though the side-chains of the HxLP motif are not involved in direct interactions with the substrate, it maintains a characteristic conformation in which Leu is an outlier in the Ramachandran plot and the peptide between Leu and Pro is in a *cis*-conformation that helps orient main-chain O atoms for substrate interactions (Figure 6 and Figure 7). Since these main-chain O atoms interact with both substrates, they appear to play a critical role in bringing the two substrates together for the catalytic reaction. In addition to these three major motifs, all members of the transcarbamylase family have a conserved Arg in the β4 strand that is involved in binding CP (R141 in human OTCase, R105 in *E. coli* ATCase, R103 in *E. faecalis* PTCase, R112 in *X. campestris* AOTCase, R110 in *B. fragilis* SOTCase and R122 in *E. coli* YTCase). The 80’s loop from an adjacent subunit also provides one or two residues involved in CP binding. However, this residue varies among different transcarbamylase members, even within the same transcarbamylase family; for example, this residue is a His in human OTCase and a Gln in *E. coli* OTCase (Table 1).

**Table 1:** Active site residues for various transcarbamylases.

Protein	CP-Binding Site	The Second Substrate-Binding Site
ATCase	S52, T53, R54, T55, R105 H134, Q137, P266, L267 Ser80 *, Lys84 *	R167, Q231, R229, L267
OTCase	S55, T56, R57, T58, R106 H133, Q136, C273, L274 R319, Q82 *	N167, D231, S235, M236, L274
PTCase	S52, T53, R54, T55, R103 H130, Q133, C269, L240 R297, Q79 *	Q164, D227, Y233, L240
AOTCase	S49, M50, R51, T52, R112 H148, Q151, C294, L295 R322, W77 *	E144, K252, L295
SOTCase	S47, L48, R49, T50, R110 H147, Q150, C274, L275 R302, W75	E142, K236, H176, R178, R278, L275
YTCase	S71, T72, R73, T74, R122 H165, Q168, C330, L331 K363, Q98 *	Q160, K270, D124, S200, K203, L331
DPTCase	S50, T51, R52, T53, R100 H128, Q131, D250, L251 K278, Q76 *	N159, T160, T211, R212, D250, L251
DBTCase	S57, T58, R59, T60, R108 H135, Q138, D271, L272 K299, Q84 *	N166, T229, S233, M234, L272
UGTCase	S74, T75, R76, T77, R126 H155, Q158, T298, L299 S102 *, K106 *	R189, S258, K261, T298, L299

* The residue is from the adjacent subunit. The residues in italics fonts indicate they are suggested substrate-binding residues that have not been confirmed by crystal structures.

In contrast to the conserved common CP binding site, different transcarbamylase members use different sets of residues to recognize their respective second substrates. In the unknotted group of transcarbamylases, the loop referred to as the 240’s loop in *E. coli* ATCase, which is equivalent to the SMG and 230’s loops in OTCase and PTCase, respectively, is involved in binding the second substrate. The RxQxER motif of the 240’s loop is found in all known ATCases. R229 and Gln231 of this motif in *E. coli* ATCase are directly involved in anchoring the β-carboxyl group of aspartate (Figure 6A). In addition to this major recognition motif, other residues such as R167 and K84 * from the adjacent subunit help to position the α-carboxyl group of aspartate. In OTCase, the SMG loop, which contains the DxxxSMG motif, is involved in recognizing the second substrate, ornithine [52,58] and D231, Ser235 and Met236 (*E. coli* OTCase numbering) are directly involved in binding ornithine (Figure 6B). Two additional residues, Asn167, and K53 in the FxKxSxRT motif, are also involved in binding ornithine. Even though K53 interacts with ornithine via a water molecule, this residue is quite conserved in OTCases, indicating the importance of this interaction. In PTCase, structure determination clearly revealed that D227 and Y233 from the equivalent 230’s loop and Q164, which hydrogen bonds to the amino group, directly shape the putrescine binding site with participation of E236 and H83 * from the adjacent subunit (Figure 6C) [29]. However, residues Y233, E236 and H83 * are not conserved in other PTCase sequences [30]. How different residues shape the putrescine binding site and whether other hypothetical PTCases with sequence variations are true PTCases remains to be established.

The second substrate recognition site in the knotted transcarbamylases (AOTCase, SOTCase and YTCase) [21,24,32] appears to be different from the unknotted group (Figure 7). In these knotted transcarbamylases, the presence of the proline-rich loop prevents the significant movement towards the active site of the equivalent 240’s loop that is involved in binding the second substrate in the unknotted transcarbamylases. Therefore, there is only one conserved lysine at the beginning of the 240’s loop, K252 in *X. campestris* AOTCase and K236 in *B. fragilis* SOTCase, that is involved in second substrate binding, in combination with the conserved glutamate residues, E144 in AOTCase and E142 in SOTCase. In *B. fragilis* SOTCase, the succinyl group of *N*-succinylornithine is anchored by three other residues: H176, R178 and R278 (Figure 7A,B) [24].

The YTCase structure clearly revealed that the enzyme is able to bind CP since all the CP binding residues can be identified and are located in positions similar to other transcarbamylases [32]. The second substrate-binding site of YTCase has some similarities to those of AOTCase and SOTCase. Residues K270 and Q160 are located at positions similar to K252 and E144 in AOTCase, and K236 and E142 in SOTCase, in order to anchor the carboxyl group of the putative substrate. Other residues such as D124, S200, K203, D334 and E344 are located around this site and may also be involved in binding this substrate (Figure 7C). The residue changes relative to SOTCase, (F112 to D124, W75 * to Q98 *), make the substrate-binding site of YTCase larger, more hydrophilic, and more negatively charged. Since YTCase likely functions as a catabolic transcarbamylase, whether the organisms that encode YTCase are able to use bulkier metabolites, such as citrulline-containing peptides as substrate, needs to be investigated.

Even though the structures of DPTCase, DBTCase and UGTCase have not yet been determined, their models can be reliably built based on the structures of OTCase and ATCase because of their sequence similarity. These structural models suggest that the D250 residue within the HDLP motif (*Saccharothrix mutabilis* DPTCase numbering), which is a characteristic feature in DPTCase and DBTCase sequences [70], will likely interact with the α-amino group of the second substrate and residue R212 from the equivalent 240’s loop may be involved in anchoring the carboxyl group of that substrate. In a similar way, T298 from the characteristic H(T/S)LP motif of UGTCase (*Rubrobacter xylanophilus* UGTCase numbering) [31] is likely to form a hydrogen bond with the ureido N atom of ureidoglycine. The exact binding mode of the substrates for these transcarbamylases will require structure determination.

## 6. Catalytic Mechanism and Domain Movement

Binding of substrates and product release are believed to be ordered in all transcarbamylases. In the anabolic transcarbamylases, CP binds before the second substrate [71] while the catabolic enzymes bind the ureido-containing substrate before phosphate [32]. The forward reaction that transfers the carbamyl group of CP to the amino group of the second substrate is thermodynamically favorable. Direct attack of the carbamyl carbon of CP by the amino group of the second substrate to form reaction products via a tetrahedral intermediate is the common catalytic mechanism for all transcarbamylases. This tetrahedral intermediate model was first proposed for *E. coli* ATCase [72]. Both the main-chain O atoms of Pro266 and Leu267 and the side-chains of Arg105, His134 and Gln137 play an important role in stabilizing the tetrahedral intermediate. When the intermediate collapses upon product formation, a proton of the amino group of the second substrate is released. Three possible acceptors of the proton have been proposed: the leaving phosphate group [72], the side-chains of Arg105, or Lys84 * of the adjacent subunit. In OTCase, the structure of *E. coli* OTCase complexed with a natural inhibitor, *N*^δ^-*N*′-sulfodiaminophosphinyl-l-ornithine models the tetrahedral intermediate. The main-chain O atoms of Cys273 and Leu274, together with the side-chains of Arg57, Thr58. Arg106, His133, and Gln136, participate in stabilizing the tetrahedral intermediate [53]. The proximity (3.1 Å) of the N^δ^ atom of ornithine to the O atom of the phosphate group is consistent with an intra-molecular proton transfer.

**Figure 8:**
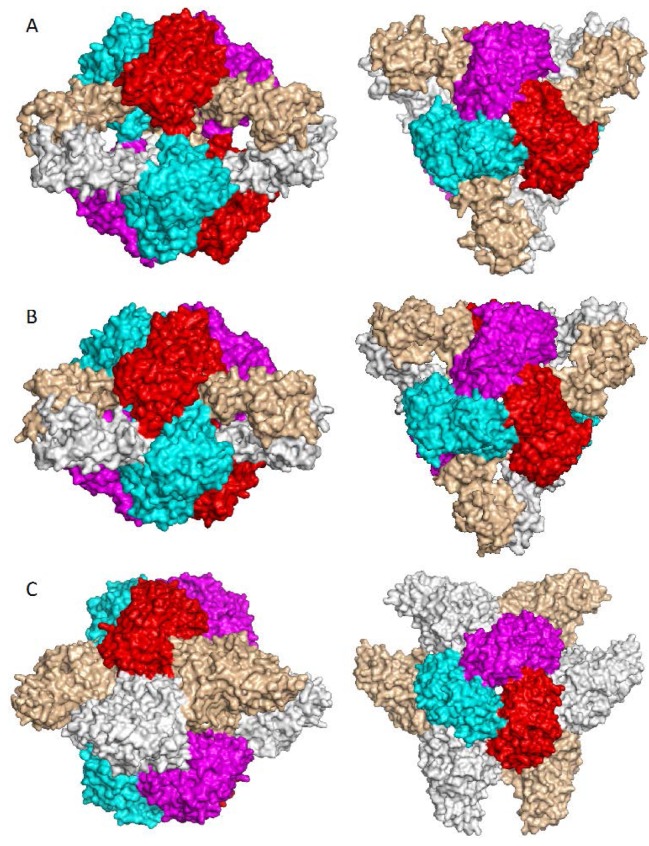
Higher oligomeric structure of ATCase. (**A**) R-state of *Escherichia coli* ATCase showing the dodecameric structure with two catalytic trimers (shown in red, magenta and cyan) at the top and bottom, and three regulatory dimers (shown in grey and tints) in the equator; (**B**) T-state of *E. coli* ATCase; (**C**) Structure of *Aquifex aeolicus* ATCase in complex with dehydroorotase. Two catalytic trimers located at the top and bottom are completely separated by three dehydroorotase dimers in the middle. **Left**: viewed perpendicular to three-fold axis; **right**: viewed down 3-fold axis.

Most structural studies of ATCase use the *E. coli* holoenzyme as a model, in which two ATCase catalytic trimers associate with three regulatory dimers to form a heterodimeric dodecameric structure [36]. Because of the restraints imposed by the regulatory subunits, the enzyme remains in the less active T (taut) state when CP binds, but the 80’s loop’s conformation changes bring S80 and K84 into the active site [71]. Subsequent aspartate binding induces conversion of the enzyme from the T state to the more active R (relaxed) state, which involves an elongation of 11 Å along the three-fold molecular axis, a relative rotation of 12° between two catalytic trimers, and a rotation of 15° for each of three regulatory dimers around their two-fold molecular axes (Figure 8A,B). Aspartate binding also induces additional conformational changes in the 80’s and 240’s loops, and relative domain closure of 8° between CP and aspartate domains. As a result, the two substrates are forced close to each other to lower the activation energy for the catalytic reaction.

Since most OTCases consist only of a catalytic trimer, the substrate induced conformation changes are not restrained by the regulatory subunits. Therefore, CP binding appears to induce most of the conformational changes of the equivalent 80’s loop and relative domain closure between CP and ornithine domains that accompany substrate binding. Ornithine binding induces the SMG loop (the equivalent 240’s loop) to swing into the active site and a small additional domain closure [60].

The proline-rich loop in members of the knotted transcarbamylase family prevents movement of the equivalent 240’s loop into the active site, making the conformational changes and relative domain movements of AOTCase associated with substrate binding much smaller (1.1°–2.2°) than those of the unknotted OTCase and ATCase [21,62].

## 7. Higher Oligomer Structure and Biological Significance

Although the isolated ATCase trimer alone has catalytic activity, ATCase often complexes or fuses with other protein units to form higher oligomer structures *in vivo*. The most known and best-characterized example is *E. coli* ATCase, a whole holoenzyme that consists of two ATCase trimers and three regulatory dimers (Figure 8A,B) [73]. This dodecameric structure is essential for the observed coupling of feedback inhibition and stimulation of catalytic activity by CTP and ATP, respectively. The higher oligomeric structure is also essential for cooperative substrate binding; the isolated catalytic trimer does not show cooperativity. Structural studies of ATCase by both X-ray crystallography and small-angle X-ray scattering (SAXS) clearly demonstrated that the ATCase holoenzyme has two different quaternary structures: the T and R states (Figure 8A,B). In the T state, the ATCase holoenzyme is constrained in its compressed quaternary structure with an open active site, low substrate affinity and low catalytic activity. Interactions between the two catalytic trimers and between catalytic chains and regulatory chains stabilize the T state. Substrate binding of both CP and aspartate shifts the structure to the R state with a closed active site and repositioning of the 80’s and 240’s loops, resulting in markedly increased substrate affinity and high catalytic activity. Nucleotide binding also alters the quaternary conformational structure of the enzyme by shifting the equilibrium between T and R states [74].

In the pyrimidine biosynthetic pathway of prokaryotes, the first three enzymes in the pathway are usually expressed by separate genes and function independently. In contrast, in mammals, these enzymes (CPS2, ATCase and DHOase), are fused together as a single polypeptide chain that self-associates to form a hexamer [75]. In *A. aeolicus*, ATCase and DHOase associate to form a dodecamer that has both ATCase and DHOase activities. The structure of the ATCase-DHOase complex reveals that the dodecamer is arranged in such a way that two ATCase trimers are located at the two polar ends with six DHOase subunits at the equator to form a hollow reactor with an internal reaction chamber that is about 60 Å in diameter (Figure 8C) [50]. All twelve active sites face the central cavity that connects to the exterior through narrow channels. Like the dodecamer of *E. coli* ATCase holoenzyme, the two ATCase catalytic trimers of the ATCase-DHOase complex have their convex faces at the polar ends of the complex and their concave sides oriented towards the central cavity. However, the two ATCase trimers are separated completely by the six DHOase subunits, in contrast to the *E. coli* holoenzyme in which there are still some interactions between the two trimers. Three features of the novel quaternary structure of the *A. aeolicus* ATCase-DHOase complex are believed to promote its biological function. (a) Direct interactions between DHOase and ATCase activate DHOase; (b) Six protein subunits form a reaction chamber to promote efficient catalytic reaction; (c) Separation of charge between the inside and the outside of the reactor helps DHOase overcome the unfavorable kinetics of condensing carbamyl-aspartate into dihydroorotate. The *A. aeolicus* ATCase- DHOase complex has been proposed as a model of the core scaffold of CAD [50]. However, the recent structural determination of the DHOase domain of human CAD raises doubts as to whether this type of assembly is feasible in CAD [76].

**Figure 9:**
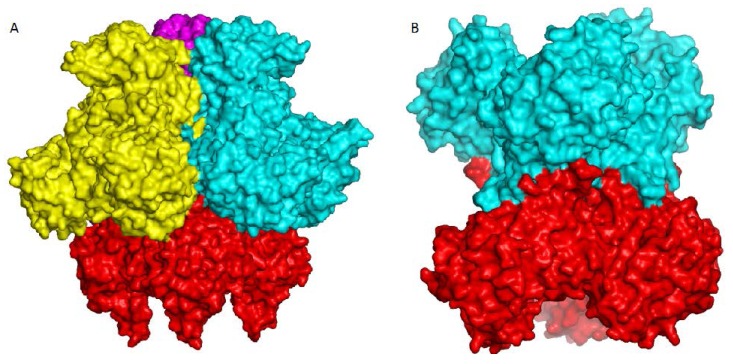
Higher oligomer structure of OTCase. (**A**) *Pyrococcus furiosus* OTCase showing tetrahedral arrangement of four catalytic trimers with concave faces outward; (**B**) *Lactobacillus hilgardii* OTCase shown the hexamer structure with convex faces interacting with each other. Different catalytic trimers are shown in different colors.

Although most catalytically active OTCases are homotrimers, higher oligomeric architectures have been reported for OTCases from thermophilic bacteria and OTCases with catabolic function. The OTCases from thermophilic bacteria, *P. furiosus* and *T. maritima*, are dodecamers arranged as a tetramer of trimers with their concave sides outwards (Figure 9A) [57,77]. The dodecameric assembly was believed to confer thermal stability of these enzymes. However, not all OTCases from thermophilic bacteria are dodecamers. For example, OTCase from *T. thermophilus* is reported to be a trimer (PDB 2EF0). The OTCases that function as catabolic enzymes *in vivo* usually assemble as larger oligomers: dodecamers for catabolic OTCases from *P. aeruginosa* and *M. panetrans*, or hexamers for the catabolic OTCase from *L. hilgardii* (Figure 9B) [19,20,55]. The larger oligomeric architectures of catabolic OTCases create additional characteristics such as strong CP homotropic cooperativity, allosteric inhibition by spermidine and activation by AMP [78]. The larger oligomeric assemblies of OTCase have two common features. (a) The concave sides of the trimer always face outwards, in contrast to the ATCase trimer in larger oligomeric structures; (b) The first helix on the convex side is involved in intertrimeric interactions [29]. There is a single report of the assembly of anabolic OTCase from *Gleobacter violaceus* as a hexamer (PDB 3GD5), but with concave sides facing inwards as in ATCase. However, the intertrimeric interactions are much weaker in this structure. Whether the biologically functioning unit is a hexamer *in vivo* remains to be established.

The functional unit of all other transcarbamylases is a homotrimer. This is expected for AOTCase and SOTCase since both of them play an anabolic role in the arginine biosynthetic pathway [21,24]. Even though PTCase and YTCase were proposed to play a catabolic role, both function as homotrimers [29,30,32]. Both PTCase and YTCase have one interesting common structural feature; the first N-terminal helix (the equivalent helix is the second helix in the YTCase structure because of the presence of an extra N-terminal helix) is covered by other helices. In PTCase, the characteristic C-terminal long helix (helix 13) covers helix 1 and it was proposed that a function of the C-terminal helix is to prevent the formation of a larger oligomer, since PTCase without the C-terminal helix will form a hexamer [29] or an even larger oligomer [30]. In YTCase, the equivalent helix is buried by the additional helices at both the N- and C-termini [32]. Whether or not the additional helices in YTCase play a role similar to the last C-terminal helix in PTCase is unknown. It would be interesting to know why these catabolic transcarbamylases develop mechanisms that prevent formation of higher oligomers.

## 8. Annotation of Transcarbamylases

The available structures of transcarbamylases demonstrate that SxRT, HPxQ and HxLP are common motifs involved in binding CP (even though some variations exist) and that these motifs are characteristic of all known transcarbamylases. Variations in four loops, the 80’s loop, 120’s loop, proline-rich loop, and 240’s loop, determine the specificity of the second substrate.

As discussed in the previous section, the transcarbamylases can be classified into two major structural groups, unknotted and knotted, based on their different folds, and two major functional groups, anabolic and catabolic, based on their different biological roles. The presence of a proline-rich loop seems to be a signature of knotted transcarbamylases. Furthermore, all currently known members of the knotted transcarbamylase group have the extended 120’s loop. Therefore, the presence of the proline-rich and extended 120’s loops can be used to distinguish knotted from unknotted transcarbamylases. Three members of the knotted group, AOTCase, SOTCase and YTCase, have been identified. The function of YTCase remains unknown, even though its structure has been determined [32]. Relative to AOTCase and SOTCase, the sequences of YTCase are longer with extra N-terminal and C-terminal helices, and an extended 240’s loop with two extra helices. However, the 80’s loop in YTCase is shorter than those of AOTCase and SOTCase and does not have a Trp that is involved in binding the second substrate. Instead, its 80’s loop is similar to those of OTCase and PTCase with Gln98 to potentially bind the substrate. YTCase can also be distinguished from AOTCase and SOTCase on the basis of their location on the chromosome. Both AOTCase and SOTCase are anabolic enzymes involved in arginine biosynthesis and their genes are usually located in the arginine biosynthetic gene cluster while YTCase is proposed to be a catabolic enzyme whose gene is close to the carbamate kinase gene in most organisms [32]. Distinguishing between AOTCase and SOTCase is more difficult because of their close sequence similarity. However, both AOTCase and SOTCase structures with substrate bound have been determined and the key residues that define their substrate specificity are clearly defined. Three residues, Glu92, Asn185 and Lys302 (*X. campestris* AOTCase numbering) can be used to distinguish AOTCase from SOTCase since the equivalent residues are A/S/P/V/Q, P and V/I/E, respectively [25].

Among members of the unknotted transcarbamylases, phylogenetic analysis divides this group into two major branches, the ATCases and OTCases [79]. PTCase, DBTCase and DPTCase belong to the OTCase branch while UGTCase belongs to the ATCase branch. Of these enzymes, structures of ATCase, OTCase and PTCase have been determined. These structures clearly demonstrate that the 240’s loops is the major site providing the second substrate specificity. DxxxSMG and RxQxxER motifs from this loop can be used to distinguish OTCase and ATCase respectively from other transcarbamylases. Even though PTCase sequences show 40%–50% sequence similarity to those of OTCase, they do not contain a specific DxxxSMG motif in the 240’s loop. Instead, (Y/W)(G/W)(V/L/I)x from the equivalent loop has been proposed to be the PTCase-specific motif [29]. Another interesting feature of the PTCase primary sequences is that the residue in the third position of Fx(E/K/N/D/A/Q)xSxRT is Gln rather than Lys in OTCase or Glu in ATCase. Additionally, PTC sequences have approximately 20 more residues at their C-terminus relative to ATCase and OTCase.

Although the structures of DPTCase and DBTCase are not available, it is expected that the 240’s loop will be involved in binding the second substrate in these enzymes because they belong to the unknotted transcarbamylase family. In this loop, the residues in the positions equivalent to the DxxxSMG OTCase recognition motif are (T/S)RWQTTG and TRWQSMG in DPTCase and DBTCase, respectively. The replacement of the conserved Asp residue in OTCase by Thr/Ser in DPTCase and DBTCase seems to be the key difference in distinguishing DPTCase and DBTCase from OTCase. Another key difference is the HxLP motif. The residue in the second position is Cys in most OTCases, whereas it is Asp in the DPTCase and DBTCase. The differences between DPTCase and DBTCase are less obvious; particularly since the DBTCase has been identified in only one species, *Streptomyces* sp., RJA2928 [70]. Whether the slight differences between (T/S)RWQTTG and TRWQSMG at the fifth and sixth position can separate these two transcarbamylases is unclear.

## 9. Future Outlook

Technological advances now allow genome sequencing at a much faster pace and lower expense, and the number of protein sequences in the database has increased exponentially. Annotating these sequences with their correct functions is a significant challenge, particularly for transcarbamylases that display only subtle differences in their primary sequences. Furthermore, new members with novel functions remain to be investigated. The transcarbamylases are involved in a wide variety of biological processes; both anabolic and catabolic, and novel transcarbamylases not yet discovered may be involved in the synthesis of natural products. Bacteria also use various ureido-containing metabolites as their energy sources for the production of ATP from arginine and agmatine by catabolic OTCase and PTCase, respectively. The discovery of UGTCase revealed that in some bacteria, a metabolite in the purine degradation pathway could be used as an energy source [31]. It may also be possible that metabolites in pyrimidine degradation pathways can also be used as energy sources in some bacteria. The possible existence of a catabolic ATCase that uses carbamyl-aspartate as an energy source or of a catabolic β-alanine transcarbamylase that uses carbamyl-β-alanine as an energy source remain to be proven. For example, the genomes of some bacteria such as the *Burkholderia* genera have two ATCase sequences. Both have typical FxExSTR and RxQxER motifs characteristic of ATCase, but one has a shorter sequence (~340 residues) and an HPGP motif, and the other has a longer sequence (~430 residues) and an HPLP motif. Whether one of these proteins plays a catabolic role is unknown. Another interesting example is *Trichomonas vaginalis* G3, which lacks the ability to synthesize many essential building blocks for DNA and protein synthesis *de novo*, particularly purines, pyrimidines and arginine [80]. *T. vaginalis* G3 obtains its energy source via fermentative metabolism under aerobic and anaerobic conditions. Four transcarbamylases in this bacterium most likely play a catabolic role in using ureido-containing compounds as an energy source to generate ATP in combination with carbamate kinase. These novel transcarbamylases could be targets for drug development against *T. vaginalis* G3 if they are proven to be essential for survival. In *T. vaginalis* G3, the arginine deiminase pathway contributes about 10% to the organism’s total energy requirement [81]. Whether the YTCase related pathway provides additional energy remains to be established.

Sequence similarities among different members of the transcarbamylase family provide many opportunities to alter substrate specificity, For example, the substrate preference of AOTCase and SOTCase can be switched by mutating a few key residues [25]. In a similar way, the substrate preference of PTCase can be changed from putrescine to ornithine by mutating the substrate recognition loop [29]. The discovery of several novel transcarbamylases further reveals that subtle differences in their primary sequences alter their substrate preferences. The substrate for UGTCase, ureidoglycine, differs in only two atoms from aspartate, the substrate for ATCase [31]. The substrates of DBTCase and DPTCase, 2,4-diaminobutyrate and 2,3-diaminoprionate, have side-chains that are one or two carbons shorter than ornithine, the substrate of OTCase [33,34,70,82]. Therefore, it is possible to engineer extant transcarbamylase members for new biological functions. Since *N*-carbamylglutamate has been used as a drug to replace *N*-acetylglutamate to activate CPS1 and restore urea cycle function in *N*-acetylglutamate synthase (NAGS) deficiency [83,84,85,86,87], it would be of great value to engineer an existing transcarbamylase such as ATCase to produce carbamylglutamate using a bacterial system. Furthermore, it might be possible to incorporate a gene to encode this novel transcarbamylase into human symbiotic bacteria such as *Lactobacilli*, already present in the small intestine [88], to allow for continuous generation of carbamylglutamate for NAGS deficiency patients.

## Supplementary Materials

Supplementary materials can be found at http://www.mdpi.com/1422-0067/16/08/18836/s1.
