# Supplementary material for: From Genome to Structure and Back Again: A Family Portrait of the Transcarbamylases
Source: Int J Mol Sci. 2015 Aug 12;16(8):18836–64. doi: 10.3390/ijms160818836 (PMC4581275; doi:10.3390/ijms160818836)
Supplement: Supplementary file 1 [file ijms-16-18836-s001.pdf]

Supplementary Information

Table S1. Structures for the members of the transcarbamylase family deposited in PDB.

Protein	Source	Note	Ligands	PDB ID	Resolution (Å)	Reference
ATCase	*E. coli*	R54A mutant	Zn(II), PALA	1ACM	2.8	[89]
		R-state	Zn(II), PAM, Malonate	1AT1	2.8	[90]
		R-state	PALA	1D09	2.01	[91]
		Catalytic trimer	PALA	1EKX	1.95	[41]
		P268A mutant T-state	Zn(II)	1EZZ	2.70	[92]
		P268A mutant R-state	PALA, Zn(II)	1F1B	2.30	[92]
		R105A mutant Catalytic trimer		1GQ3	2.01	
		R105A mutant	PALA, Zn(II)	1I5O	2.80	[93]
		T82A mutant	Malic acid, Zn(II)	1NBE	2.60	[94]
		R-state	PALA, Zn(II)	1Q95	2.46	[95]
		R-state	Citrate, Zn(II), Phosphate	1R0B	2.90	[95]
		T-state	Zn(II), Phosphate, NCD	1R0C	2.37	[96]
		T-state	CTP	1RAA, 1RAB, 1RAC, 1RAD, 1RAE, 1RAF, 1RAG, 1RAH, 1RAI	2.50	[97]
		K244N mutant	Malonate, Zn(II)	1SKU	2.60	[98]
		E50A mutant	PALA, Zn(II)	1TTH	2.80	[99]
		E50A mutant	PAM, Zn(II)	1TU0	2.55	[99]
		E50A mutant	PAM, Malonate, CTP, Zn(II)	1TUG	2.10	[99]

Table S1. *Cont.*

Protein	Source	Note	Ligands	PDB ID	Resolution (Å)	Reference
		Q137A mutant	PALA, Zn(II)	1XJW	2.71	[100]
			CTP, Zn(II)	1ZA1	2.20	[71]
			CTP, CP, Zn(II)	1ZA2	2.50	[71]
		D236A mutant	PAM, Zn(II)	2A0F	2.90	[101]
			PAM, Malonate, Zn(II)	1AT1	2.80	[90]
			Zn(II)	2ATC	3.00	[102]
			PAM, Zn(II)	3AT1	2.80	[90]
		Catalytic trimer		3CSU	1.88	[40]
			ATP, Zn(II)	4AT1	2.60	[103]
			CTP, Zn(II)	5AT1	2.60	[103]
			Zn(II)	6AT1	2.60	[103]
			PAM, Malonate, ATP, Zn(II)	7AT1	2.80	[103]
			PAM, Malonate, CTP, Zn(II)	8AT1	2.80	[103]
			PALA, Zn(II)	8ATC	2.50	[104]
			Zn(II)	9ATC	2.40	[105]
			CP, Alanosine	2AIR	2.00	[106]
		T-state	CTP, EOA	2FZC	2.46	[107]
		T-state	CTP, EOB	2FZG	2.25	[107]
			CTP, EOZ	2FZK	2.50	[107]
		R-state	PALI	2IPO	2.60	[108]
		R-state	PALI	2H3E	2.30	[109]
		R-sate D236A	PAM, aspartate	2HSE	2.60	Unpublished

Table S1. *Cont.*

Protein	Source	Note	Ligands	PDB ID	Resolution (Å)	Reference
		rH20A		2QGF	2.20	[110]
		rD19A		2PG9	2.70	[110]
		pH = 8.5		3D7S	2.80	[111]
		R-state	ATP	4KGV	2.10	[112]
		R-state	CTP	4KGX	2.20	[112]
		R-state	ATP, Mg^{2+}	4KH0	2.25	[112]
			dCTP	4FYV	2.10	[113]
			CTP	4FYX	2.10	[113]
			dCTP, CTP, Mg^{2+}	4FYW	2.10	[113]
		R-state	UTP	4F04	2.30	[114]
		K164E/E239K		4E2F	2.80	[115]
		C47A/A241C		3NPM	2.10	[116]
		Natural source		4WTO	2.30	Unpublished
		C47A/A241C		3MPU	2.86	[116]
	M. jannaschii	Regulatory dimer	ATP	2YWW	2.00	Unpublished
		Catalytic trimer		2RGW		[46]
		Catalytic trimer		3E2P	3.0	[45]
				4EKN	2.50	[47]
	M. profunda	V2A T-state		2BE7	2.85	[48]
	P. abyssi	Catalytic trimer	PALA	1ML4	1.80	[42]
	S. acidocaldarius	T-state	Zn(II)	1PG5	2.60	[43]
		T-state	CTP	2BE9	2.60	[44]
	A. aeolicus	Complexed with DHO		3D6N	2.3	[50]
		Complexed with DHO	PALA	4BJH	2.2	[51]
	B. subtilis	Catalytic trimer		2AT2	3.0	[7]
				3R7D	2.2	[49]
			CP	3R7F	2.10	[49]
			PALA	3R7L	2.59	[49]
	T. cruzi	Catalytic trimer		4IV5	2.10	Unpublished
	Y. pestis	Catalytic trimer		3LXM	2.0	Unpublished
aOTCase	*E. coli*	Trimer		1AKM	2.8	[54]
			Psorn	1DUV	1.7	[53]
			PALO	2OTC	2.80	[52]
	Human	Trimer	CP, Norvaline	1C9Y	1.90	[117]
			CP	1EP9	2.40	[60]

Table S1. *Cont.*

Protein	Source	Note	Ligands	PDB ID	Resolution (Å)	Reference
			CP	1FVO	2.60	[60]
			PALO	1OTH	1.85	[58]
	Ovine	Trimer		1FB5	3.50	[61]
	B. anthracis	Trimer	CP, norvaline	4NF2	1.74	Unpublished
				4EP1	3.25	Unpublished
	V. vulnificus	Trimer		4KWT	1.86	Unpublished
		Trimer	Pi, Citrulline	4JQO	2.08	Unpublished
			CP, arginine	4JHX	1.85	Unpublished
			CP	4JFR	2.17	Unpublished
			CP, norvaline	4H31	1.70	Unpublished
				3UPD	2.90	Unpublished
	T. thailandensis		Pi	4F2G	2.10	[118]
	Py. furiosus	Dodecamer	Phosphate	1A1S	2.70	[57]
				1PVV	1.87	[77]
	T. maritima	Dodecamer	Phosphate	1VLV	2.25	Unpublished
	T. thermophilus	Trimer		2EF0	2.0	Unpublished
	M. tuberculosis	Trimer	CP<br>Norvaline	2I6U	2.2	[119]
		Trimer		2P2G	2.7	[119]
	G. lamblia	Trimer	PALO	3GRF	2.0	[120]
	C. jejuni	Trimer		3TPF	2.70	[121]
	C. immitis			3SDS	2.80	[122]
	B. melitensis	Trimer		4OH7	1.50	Unpublished
	G. violaceus	hexamer		3GD5	2.10	Unpublished
cOTCase	*Ps. aeruginosa*	Dodecamer	Phosphate	1DXH	2.50	Unpublished
				1ORT	3.0	[55]
	M. penetrans	Dodecamer		4AMU	2.5	[19]
				4ANF	2.6	[19]
	L. hilgardii	Hexamer		2W37	2.1	[20]
AOTCase	*X. campestris*	Trimer	Sulfate	3KZC	2.20	[21]
			ACT,<br>Sulfate	3KZK	1.90	[21]
			CP	3KZM		[62]
			AORN	3KZN		[62]
			CP,<br>ANOR	3KZO		[62]
		E92A	CP,<br>SNOR	3L02	2.30	[25]
		E92P	CP, SNOR	3L04	2.50	[25]
		E92S	CP,<br>SNOR	3L05	2.80	[25]

Table S1. *Cont.*

Protein	Source	Note	Ligands	PDB ID	Resolution (Å)	Reference
		E92V	CP, SNOR	3L06	2.81	[25]
			PALAO	3M4J	2.2	[123]
		K302A	PALAO	3M4N	1.9	[123]
		K302E	PALAO	3M5C	1.85	
SOTCase	*B. fragilis*	Trimer	Phosphate	1JS1	2.0	[23]
			Sulfate, SNOR	2FG6	2.80	[24]
			CP, SNOR	2FG7	2.90	[24]
		P90E	CP, ANOR	2G7M	2.90	[25]
PTCase	*E. faecalis*	R54G	Guanidinine	4AM8	1.99	Unpublished
		Trimer	PALP	4A8H	2.50	[29]
			PALO	4A8P	2.00	[29]
		Hexamer	PALO	4A8T	1.59	[29]
		Trimer		3TXX	3.20	[30]
YTCase	*E. coli*	Trimer		3Q98	2.0	[32]
	E. faecalis	Trimer		2YFK	2.55	Unpublished
			CP	2YFF		Withdrawn
			CP + Taurine	2YFM		Withdrawn
